# Supplementary material for: Time of night and moonlight structure vertical space use by insectivorous bats in a Neotropical rainforest: an acoustic monitoring study
Source: PeerJ. 2020 Dec 18;8:e10591. doi: 10.7717/peerj.10591 (PMC7751414; doi:10.7717/peerj.10591)
Supplement: Supplemental Information 1 [file peerj-08-10591-s001.pdf]

Supplemental file for: Acoustic monitoring shows temporal variation in vertical stratification of neotropical rainforest bats

Dylan G.E. Gomes, Giulliana Appel, and Jesse R. Barber

Model diagnostics for 'all bats' model

All bats were included in a model... describe model, which is displayed in Figures 1 and 2 in the main text. Below are some diagnostic plots for this model.

Rhat ($\hat{R}$), also known as the Gelman-Rubin statistic, values should be near 1. This visual shows that indeed all $\hat{R}$ values are near 1, thus the chains converged.

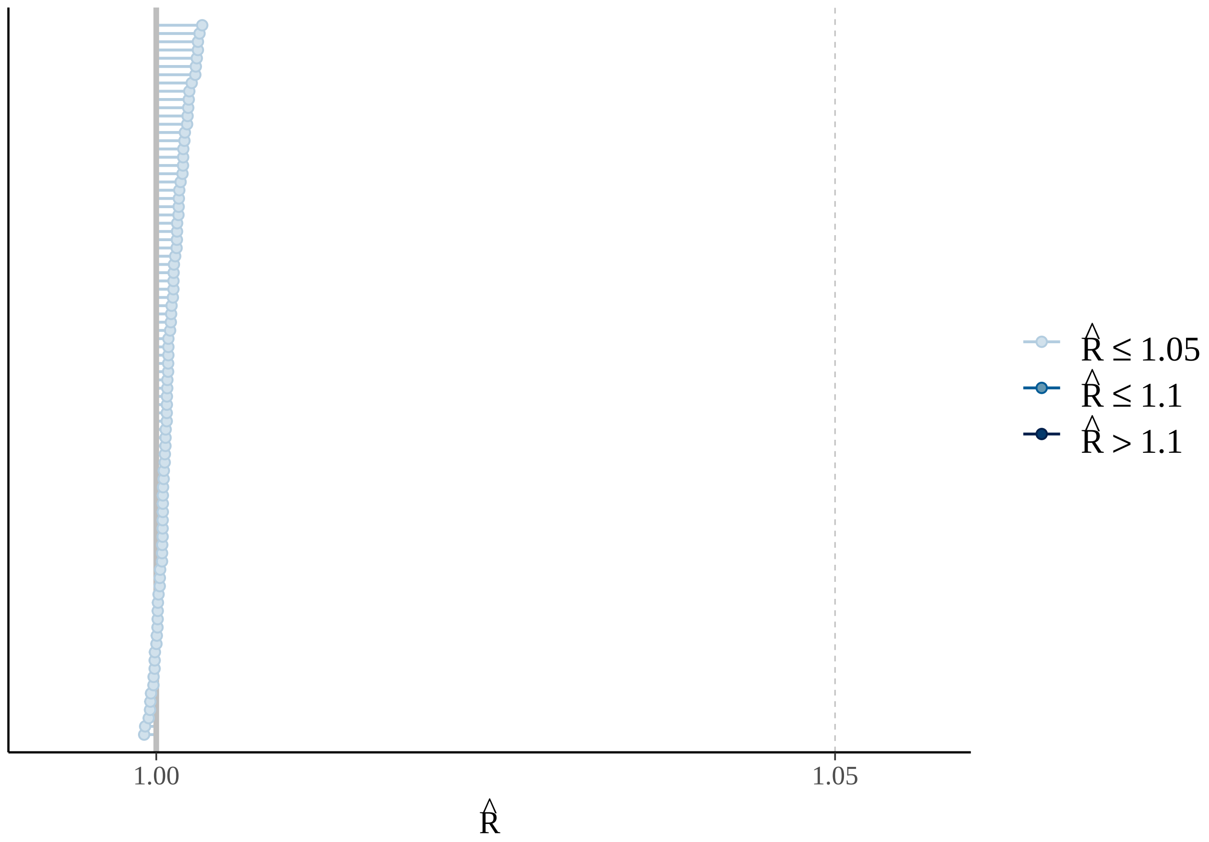

Collinearity

Variance Inflation Factors (VIF) are used to assess collinearity. Here we see that there is no collinearity - they are all below 2 (sometimes 10 is an arbitrary cutoff for when one should be concerned).

```
## # Check for Multicollinearity
##
## Low Correlation
##
##       Parameter  VIF Increased SE
##  scale(M.Illm) 1.01         1.00
##            HOUR 1.40         1.18
##          Canopy 1.02         1.01
##     HOUR:Canopy 1.38         1.18
```

Trace plots

Trace plots below are for `above canopy` model coefficients by bat species or acoustic complex. All trace plots show that the chains are well mixed.

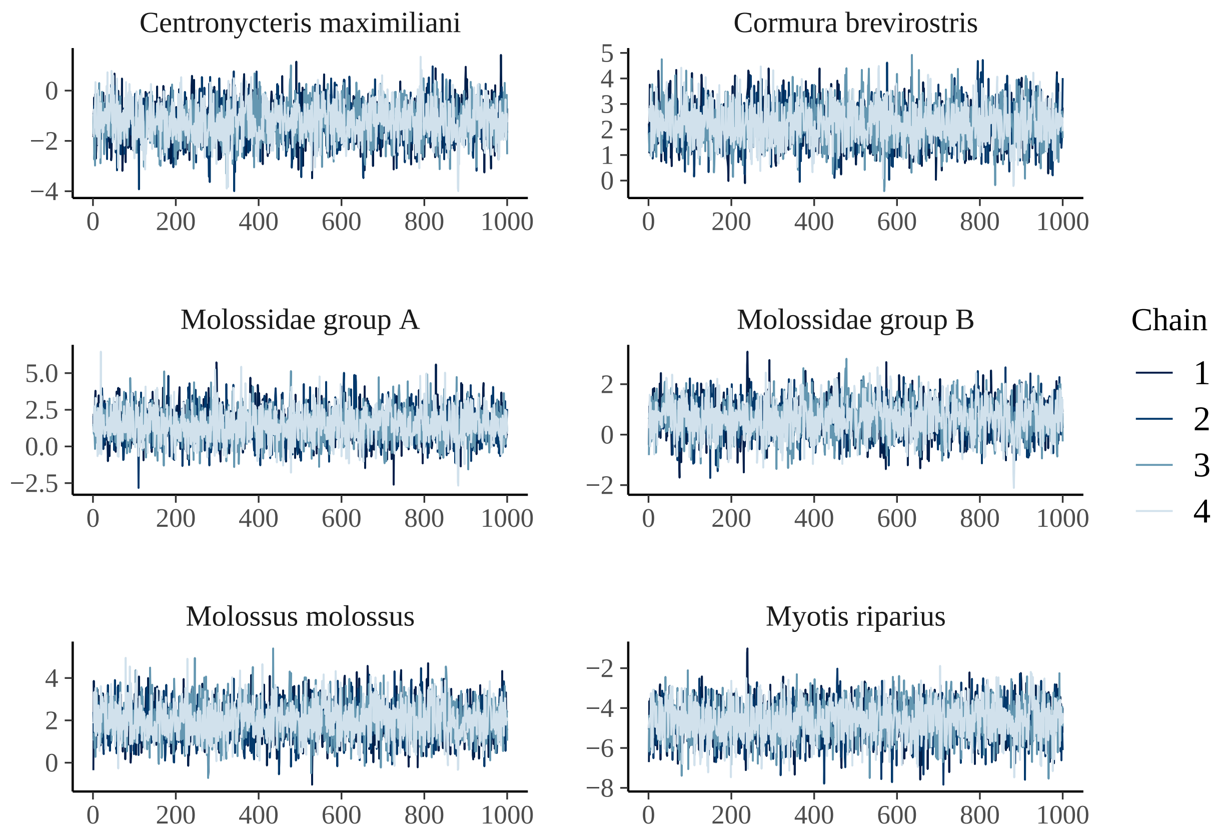

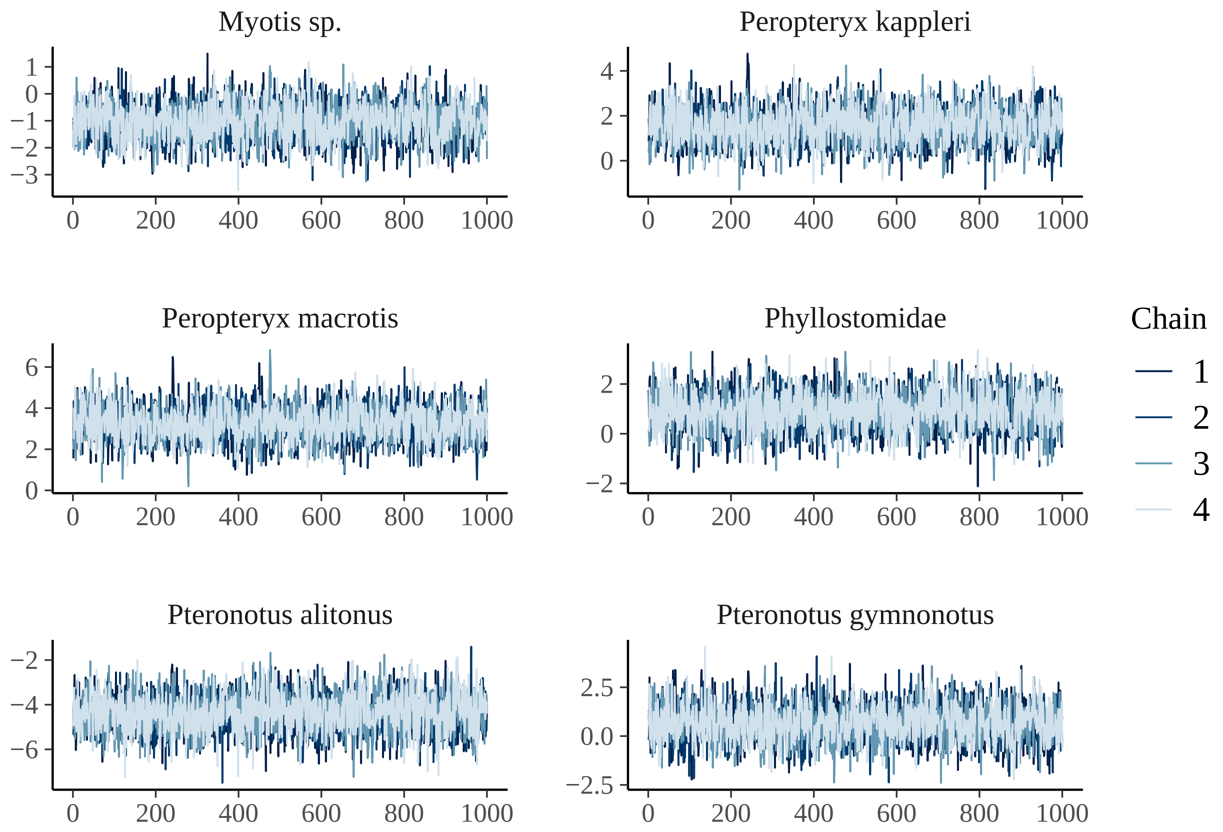
Myotis sp.
Peropteryx kappleri
Peropteryx macrotis
Phyllostomidae
Pteronotus alitonus
Pteronotus gymnonotus
Chain
1
2
3
4
0
200
400
600
800
1000

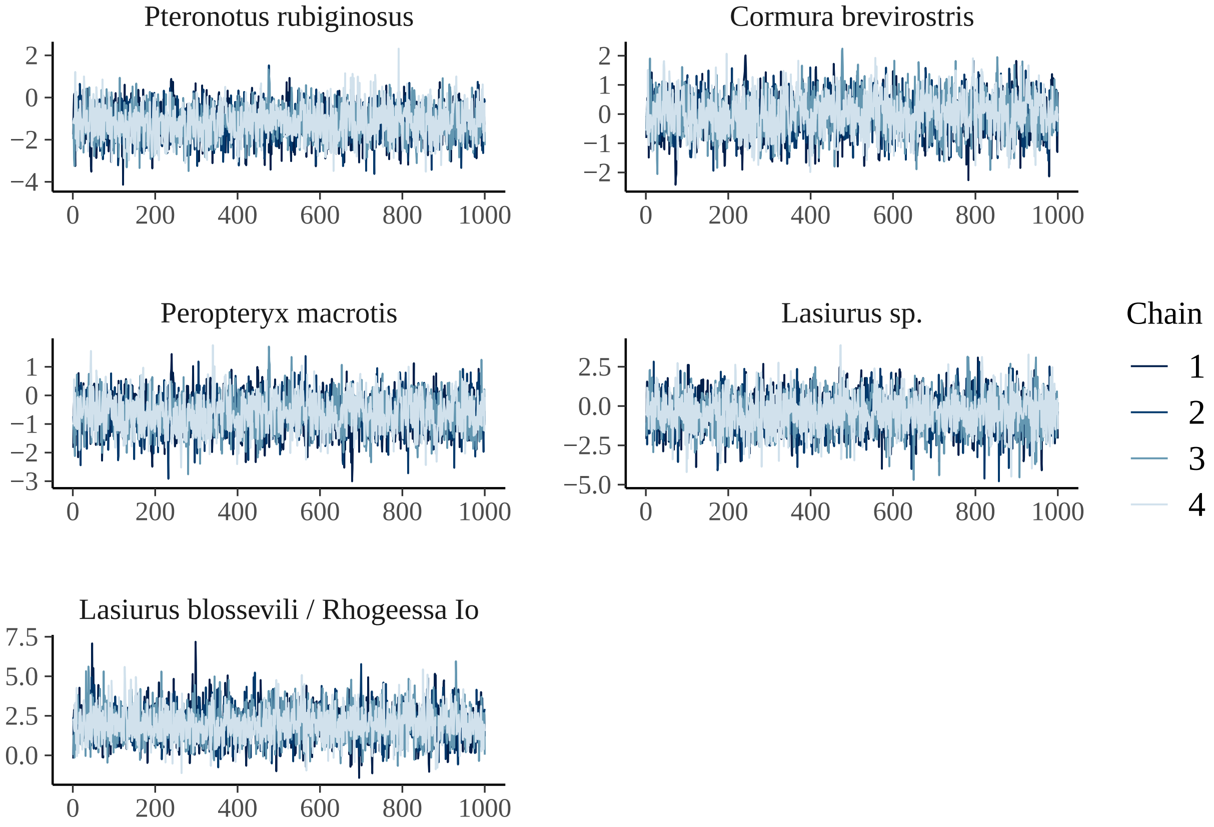
Pteronotus rubiginosus
Cormura brevirostris
Peropteryx macrotis
Lasiurus sp.
Lasiurus blossevili / Rhogeessa Io
Chain
1
2
3
4
0
200
400
600
800
1000

Zero-inflation

Count data can sometimes be zero-inflated. Here we visualize whether the actual proportion of zeros (solid vertical line) is within a possible range of zeros given by simulated data from our model. Here we see that the actual numbers of zeros are well within the range of possibilities given our model.

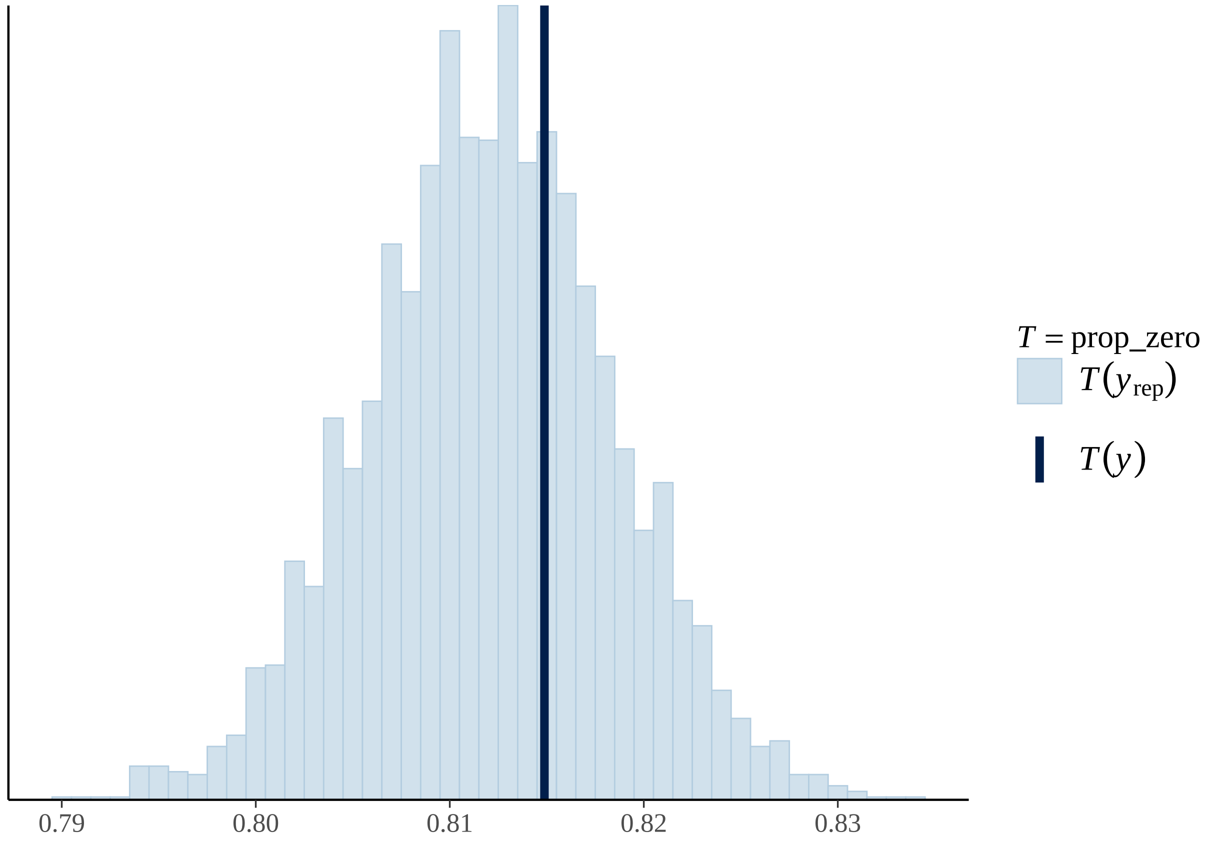

Kernel density estimates for individual species not included in main text

All kernel density estimate (see text and code for description) plots have raw occurrences plotted at top and bottom of plot, which are matched by color to density estimate and sample size (found in plot subtitle). Data from above the canopy are coded blue and data from below the canopy are coded brown. Dashed kernel density plots indicate that the reader should interpret with caution: low sample size. An additional point of caution: note that acoustic complexes are likely mixes of different species. Thus, it is not appropriate to assume that patterns from an acoustic complex would match the pattern from an individual species within that complex.

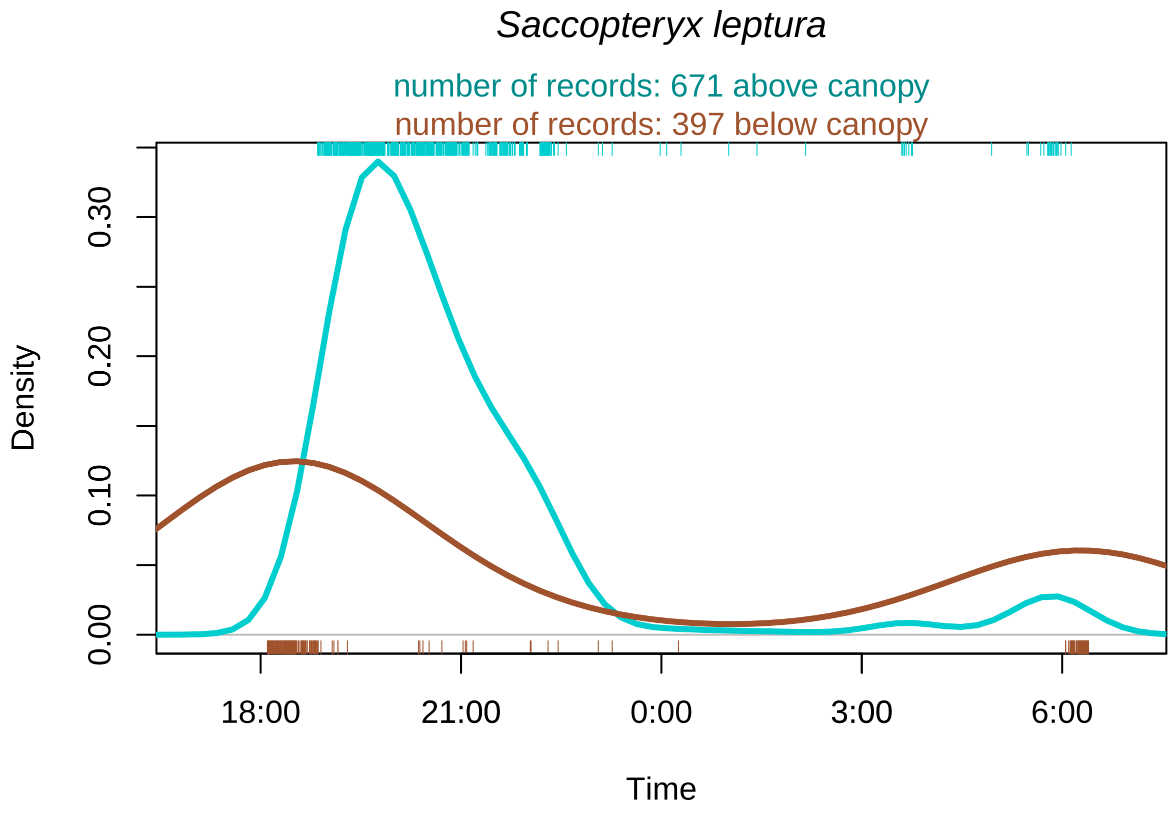

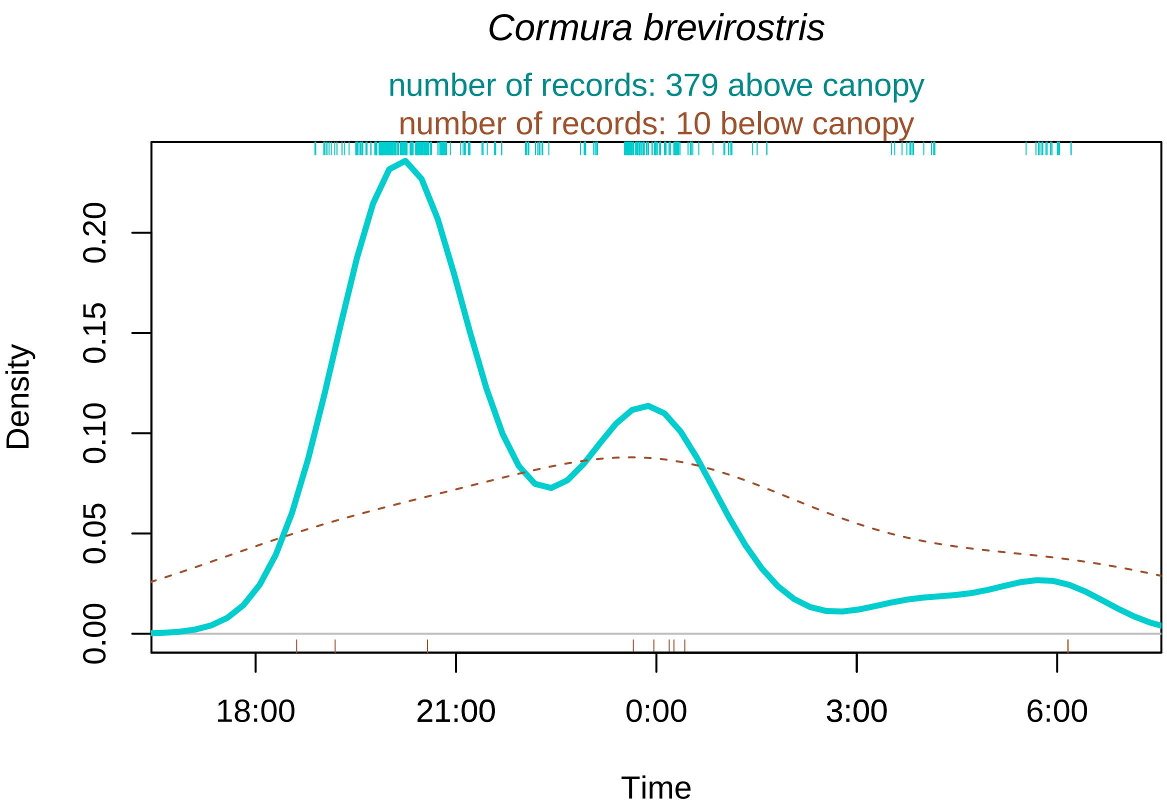

Cormura brevirostris
number of records: 379 above canopy
number of records: 10 below canopy
Density
0.00
0.05
0.10
0.15
0.20
18:00
21:00
0:00
3:00
6:00
Time

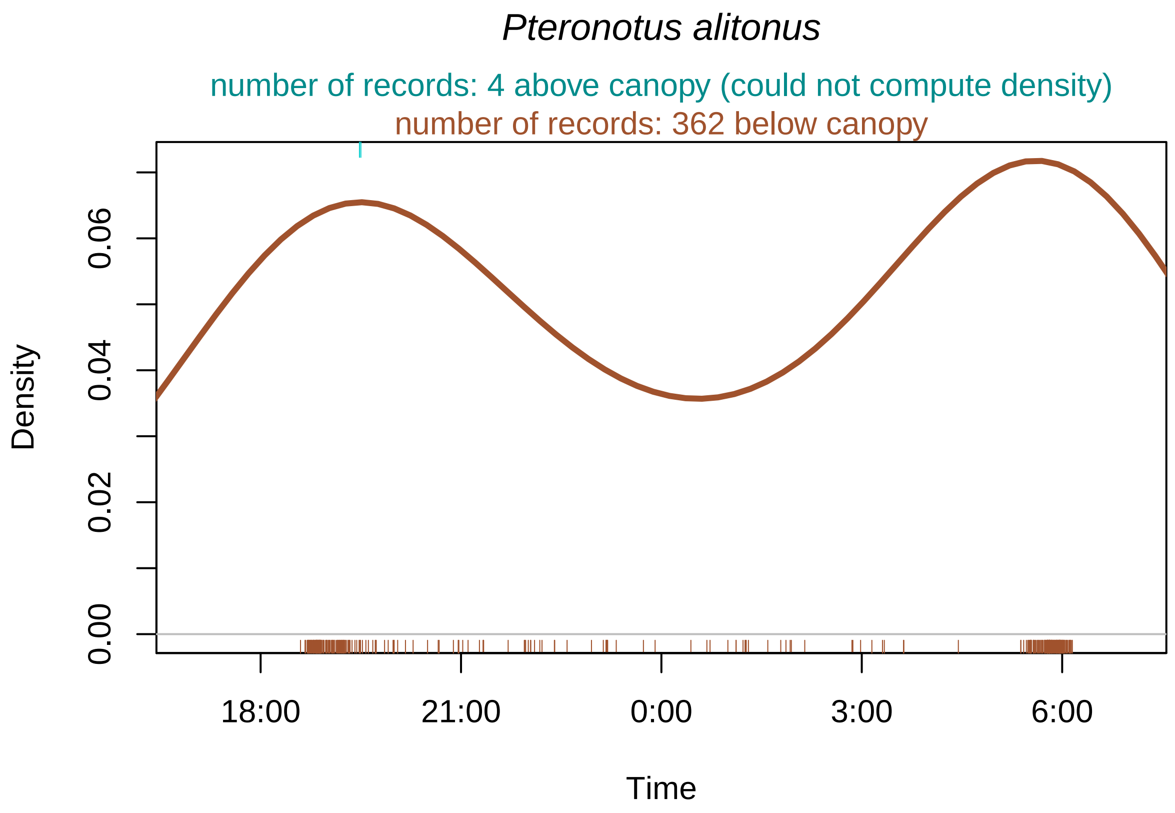
Pteronotus alitonus
number of records: 4 above canopy (could not compute density)
number of records: 362 below canopy
Density
0.00
0.02
0.04
0.06
18:00
21:00
0:00
3:00
6:00
Time

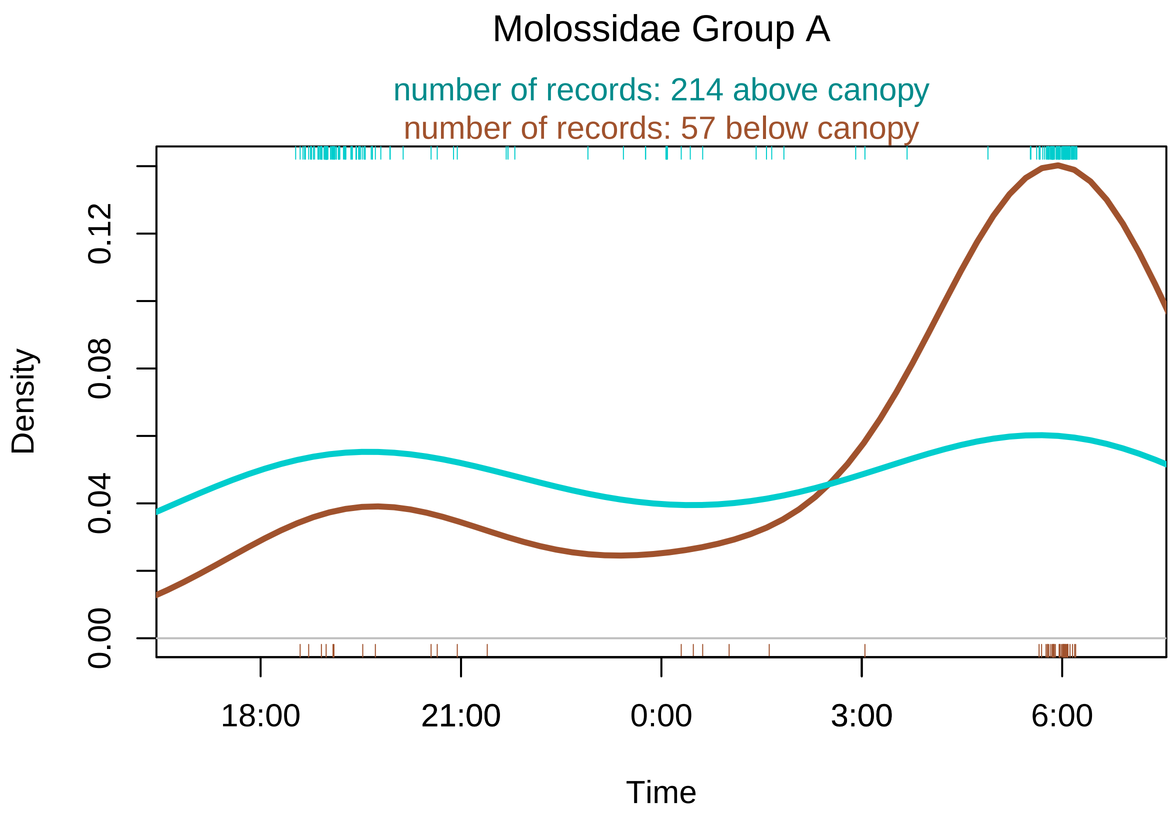

Molossidae Group A
number of records: 214 above canopy
number of records: 57 below canopy
Density
0.00
0.04
0.08
0.12
18:00
21:00
0:00
3:00
6:00
Time

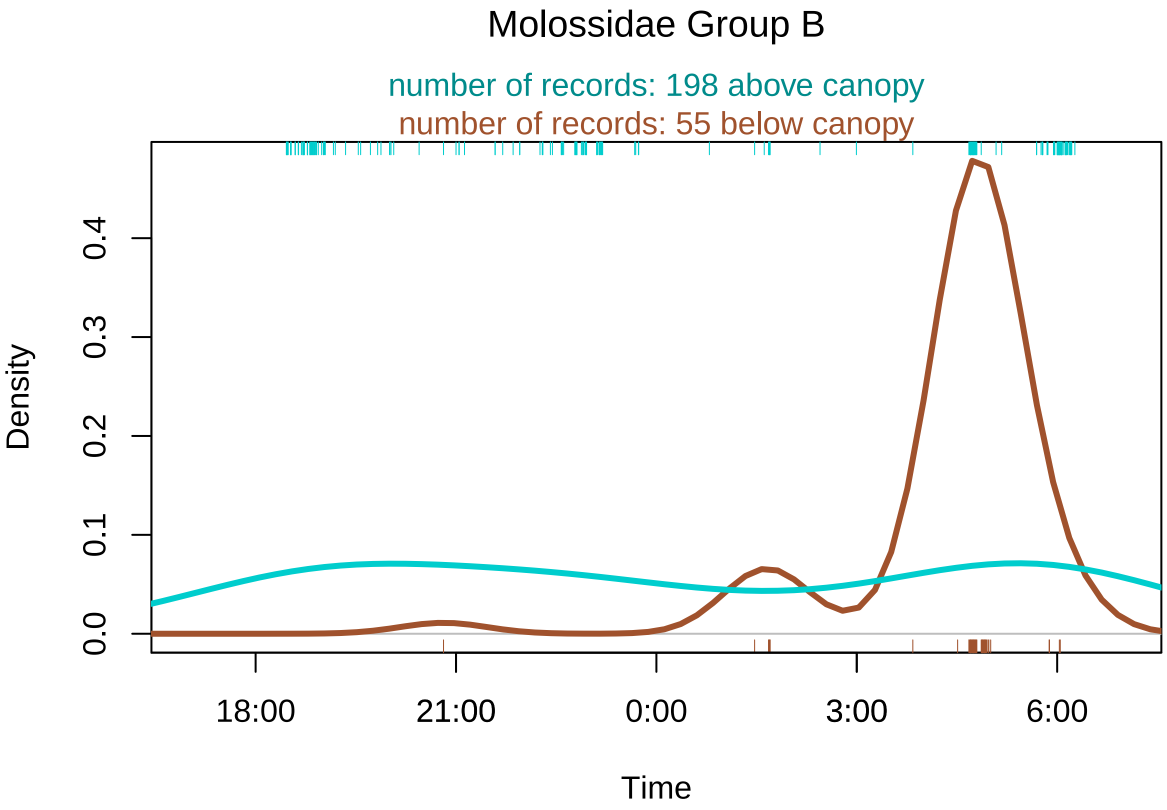

Molossidae Group B
number of records: 198 above canopy
number of records: 55 below canopy
Density
0.0
0.1
0.2
0.3
0.4
18:00
21:00
0:00
3:00
6:00
Time

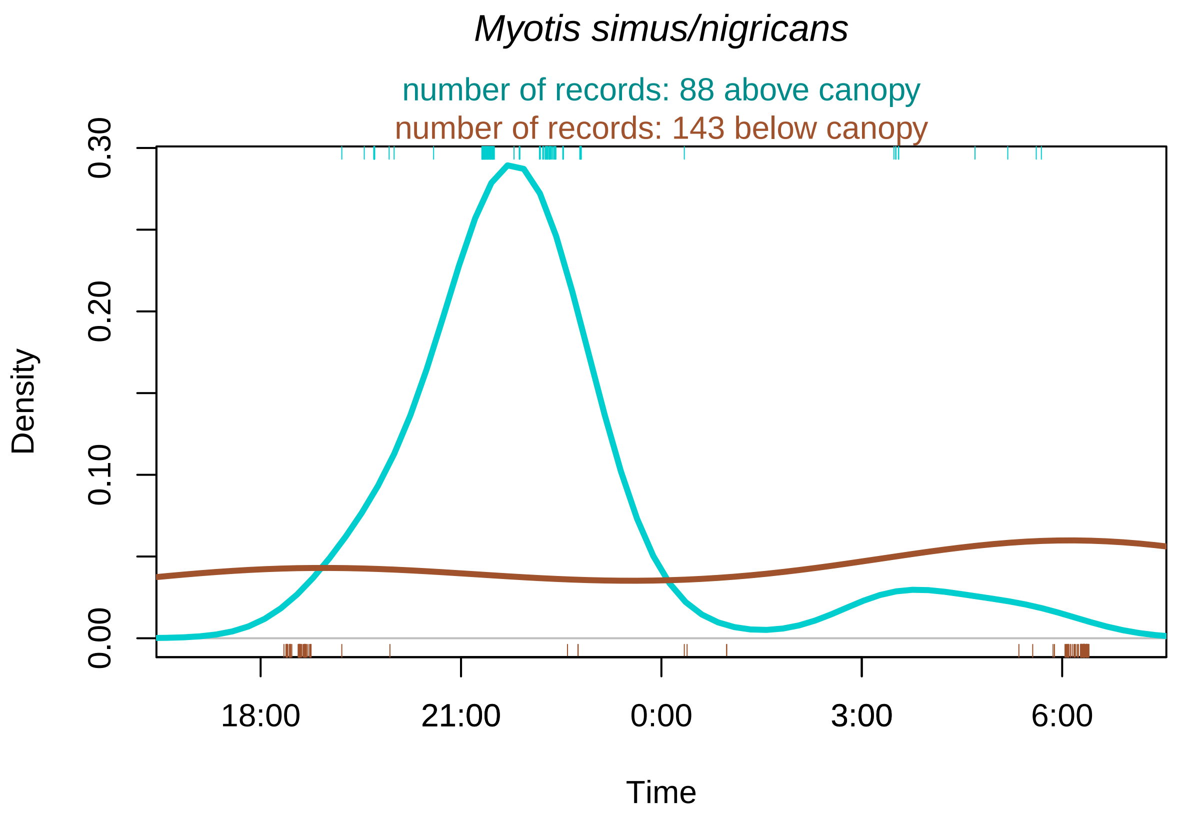
Myotis simus/nigricans
number of records: 88 above canopy
number of records: 143 below canopy
Density
0.00
0.10
0.20
0.30
18:00
21:00
0:00
3:00
6:00
Time

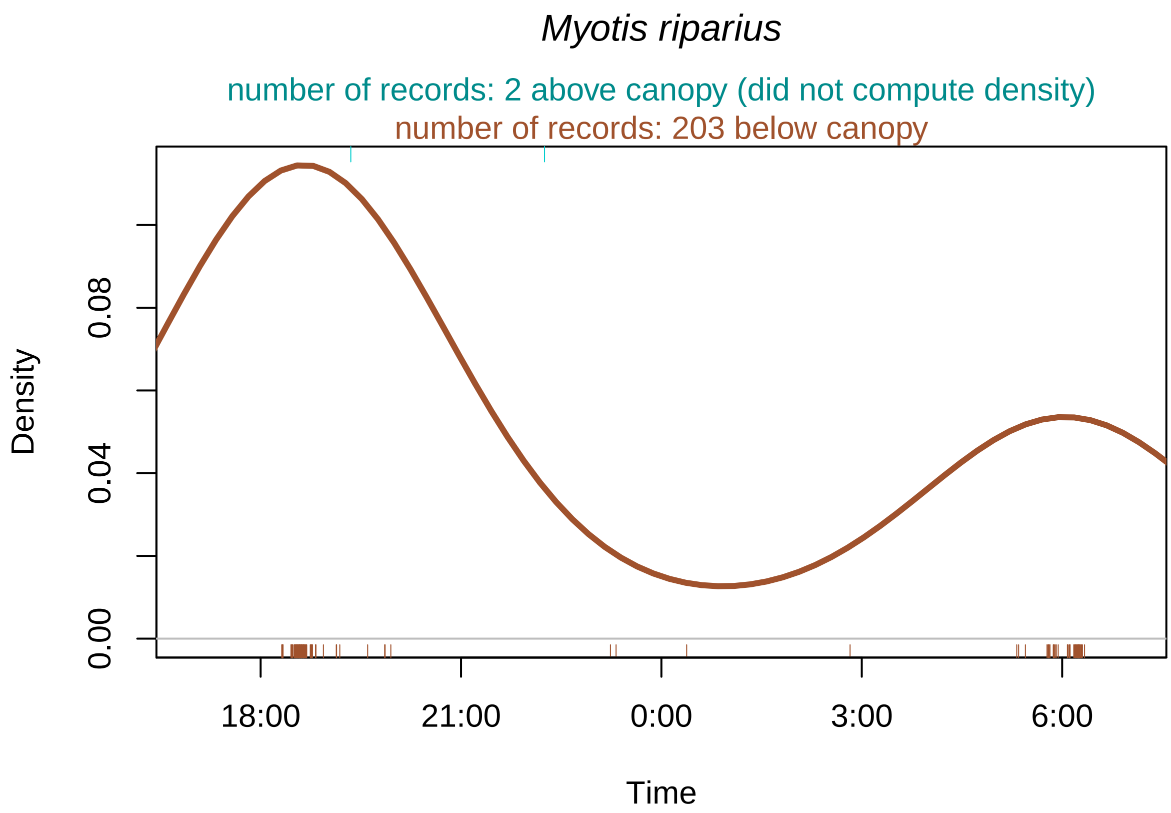

Myotis riparius
number of records: 2 above canopy (did not compute density)
number of records: 203 below canopy
Density
0.00
0.04
0.08
18:00
21:00
0:00
3:00
6:00
Time

Wing loading and apsect ratio visualizations by fixed effect estimates

Model estimates (fixed effects) are plotted against wing aspect ratios and wing loading, respectively, for 8 species that were available from Núñez et al. (2019). In the case where Núñez et al. (2019) offered wing loading / wing aspect ratio values from multiple sources, we took the average of those values for the visualization. A simple linear model with standard errors from the function `geom_smooth` within `ggplot2` (Wickham 2011) is plotted through the data points. However, as there are very few data in these plots, we caution that they should be interpreted as exploratory visualizations, and not statistical tests.

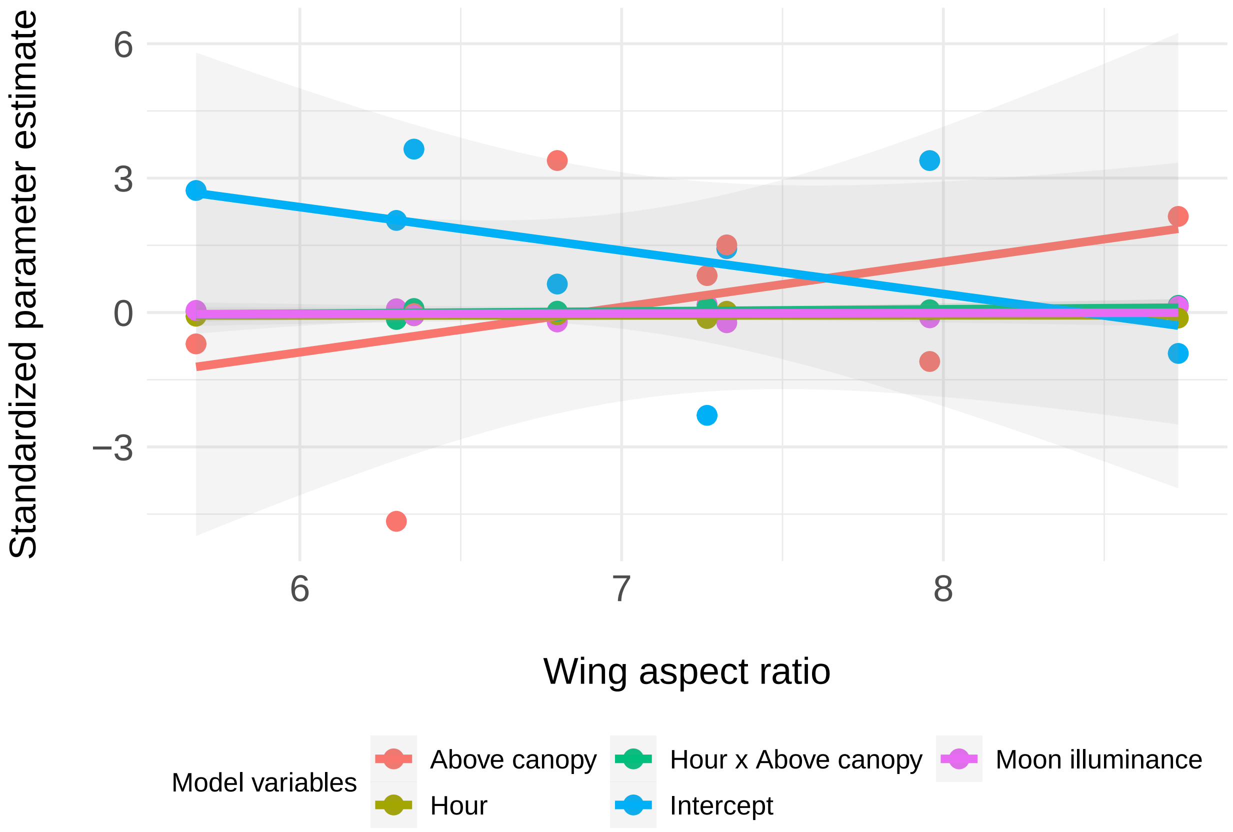

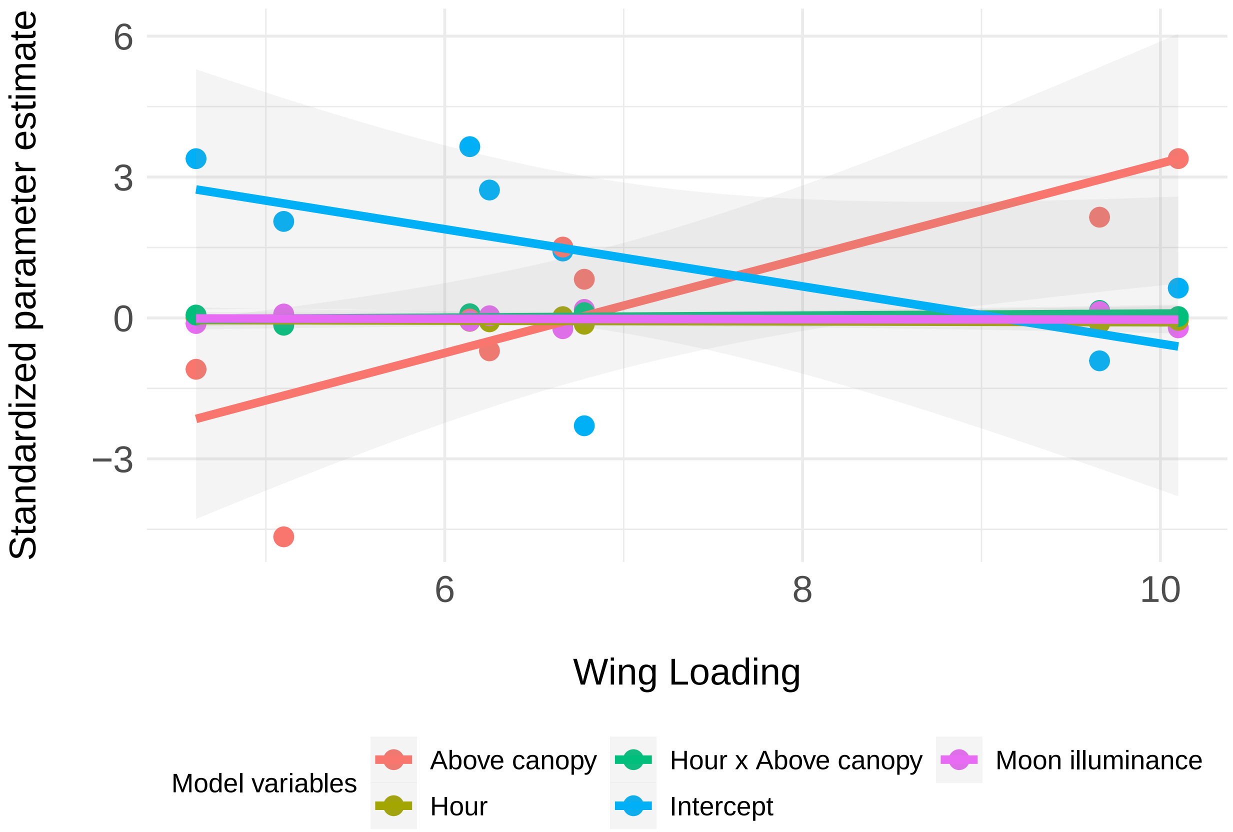
Standardized parameter estimate
6
3
0
−3
6
8
10
Wing Loading
Model variables
Above canopy
Hour x Above canopy
Moon illuminance
Hour
Intercept

Wing loading and apsect ratio visualizations by moonlight only

Model estimates for moonlight illuminance plotted against wing aspect ratios and wing loading, respectively, for 8 species that were available from Núñez et al. (2019). These data are the same as above, but with all other estimates omitted from the visualization. This allows us to more clearly see the relationship that wing morphometrics (and thus foraging strategy) might have (or not have) with moonlight illuminance. Here, there are no apparent relationships. However, as there are very few data in these plots, we caution that they should be interpreted as exploratory visualizations, and not statistical tests.

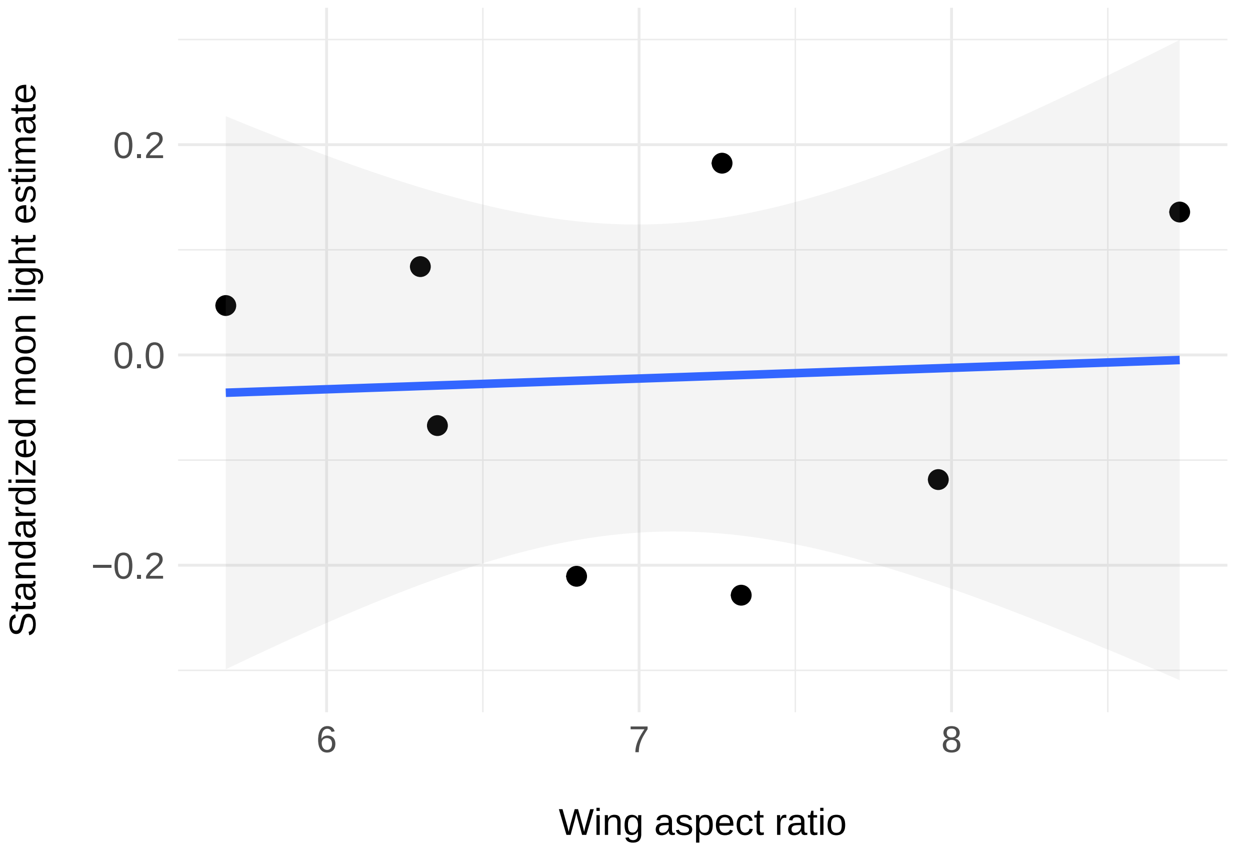

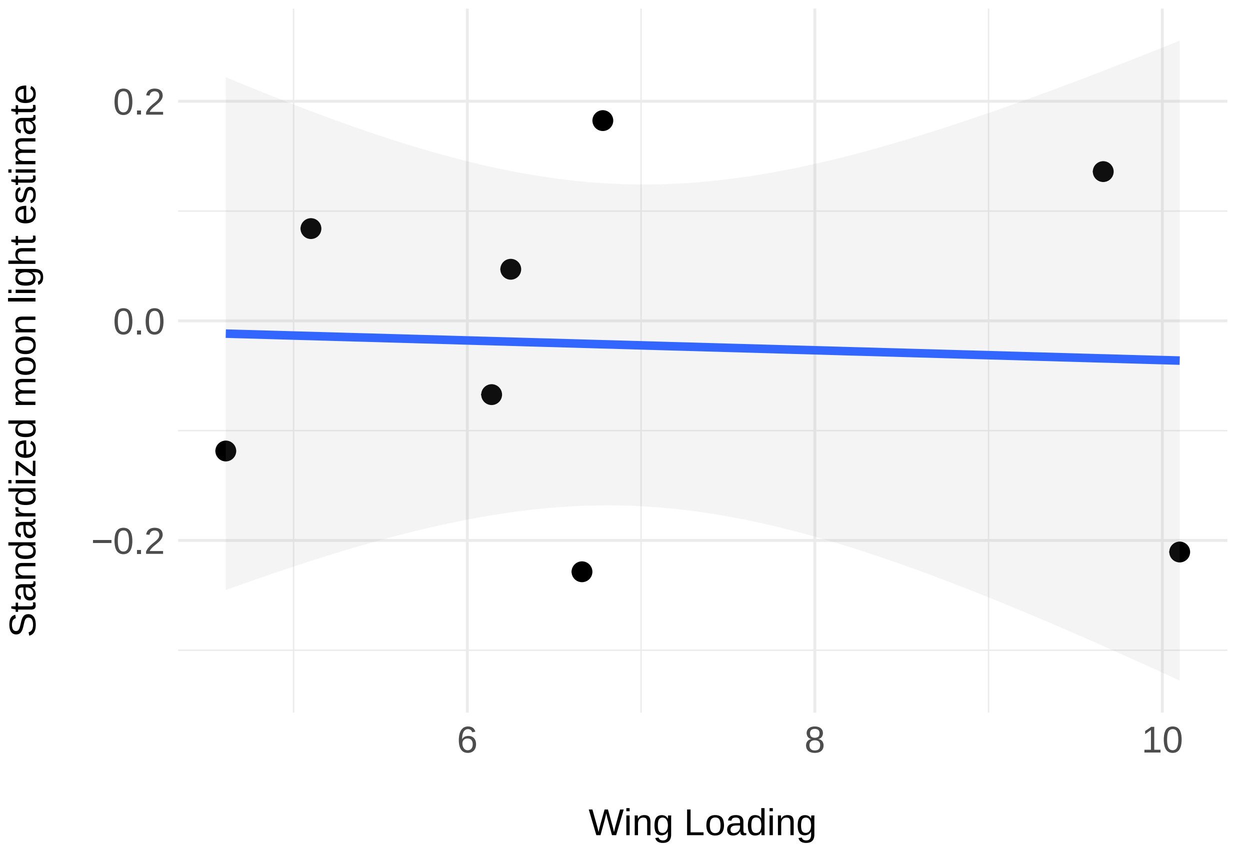

References

Núñez, S.F., Baucells, A.L., Rocha, R., Farneda, F.Z., Bobrowiec, P.E., Palmeirim, J.M. and Meyer, C.F., 2019. Echolocation and wing morphology: key trait correlates of vulnerability of insectivorous bats to tropical forest fragmentation. Frontiers in Ecology and Evolution, 7, p.373.

Wickham, H., 2011. ggplot2. Wiley Interdisciplinary Reviews: Computational Statistics, 3(2), pp.180-185.
